# Multi-Institutional Study Validates Safety of Intraoperative Cesium-131 Brachytherapy for Treatment of Recurrent Head and Neck Cancer

**DOI:** 10.3389/fonc.2021.786216

**Published:** 2021-11-26

**Authors:** Adam Luginbuhl, Alyssa Calder, David Kutler, Chad Zender, Trisha Wise-Draper, Jena Patel, Michael Cheng, Vidhya Karivedu, Tingting Zhan, Bhupesh Parashar, Shuchi Gulati, Min Yao, Pierre Lavertu, Vinita Takiar, Alice Tang, Jennifer Johnson, William Keane, Joseph Curry, David Cognetti, Voichita Bar-Ad

**Affiliations:** ^1^ Department of Otolaryngology, Thomas Jefferson University, Philadelphia, PA, United States; ^2^ Department of Otolaryngology, Weill Cornell Medical Center, New York, NY, United States; ^3^ Department of Otolaryngology, University of Cincinnati Medical Center, Cincinnati, OH, United States; ^4^ Department of Medical Oncology, University of Cincinnati Medical Center, Cincinnati, OH, United States; ^5^ Department of Medical Oncology, Ohio State University, Columbus, OH, United States; ^6^ Division of Biostatistics, Department of Pharmacology and Experimental Therapeutics, Thomas Jefferson University, Philadelphia, PA, United States; ^7^ Department of Radiation Oncology, Zucker School of Medicine at Hofstra/Northwell, New York, NY, United States; ^8^ Department of Radiation Oncology, University Hospitals Cleveland Medical Center Seidman Cancer Center, Cleveland, OH, United States; ^9^ Department of Radiation Oncology, University of Cincinnati Medical Center, Cincinnati, OH, United States; ^10^ Department of Medical Oncology, Thomas Jefferson University, Philadelphia, PA, United States; ^11^ Department of Radiation Oncology, Thomas Jefferson University, Philadelphia, PA, United States

**Keywords:** head and neck cancer, recurrent, surgery, brachytherapy, Cesium-131, reirradiation head and neck

## Abstract

**Introduction:**

Surgery is the primary treatment for resectable, non-metastatic recurrent head and neck squamous cell carcinoma (HNSCC). We explore the safety and oncologic benefit of intraoperative Cesium-131 (Cs-131) brachytherapy combined with salvage local and/or regional surgical resection.

**Methods and Materials:**

Findings were reported from a single arm multi-institutional prospective phase 1/2 trial involving surgery plus Cs-131 (surgery + Cs-131) treatment. The results of two retrospective cohorts—surgery alone and surgery plus intensity modulated radiation therapy (surgery + ReIMRT)—were also described. Included patients had recurrent HNSCC and radiation history. Safety, tumor re-occurrence, and survival were evaluated.

**Results:**

Forty-nine patients were enrolled in the surgery + Cs-131 prospective study. Grade 1 to 3 adverse events (AEs) occurred in 18 patients (37%), and grade 4 AEs occurred in 2 patients. Postoperative percutaneous endoscopic gastrostomy (PEG) tubes were needed in 10 surgery + Cs-131 patients (20%), and wound and vascular complications were observed in 12 patients (24%). No cases of osteoradionecrosis were reported in the surgery + Cs-131 cohort. We found a 49% 2-year disease-free survival at the site of treatment with a substantial number of patients (31%) developing metastatic disease, which led to a 31% overall survival at 5 years.

**Conclusions:**

Among patients with local/regional recurrent HNSCC status-post radiation, surgery + Cs-131 demonstrated acceptable safety with compelling oncologic outcomes, as compared to historic control cohorts.

**Clinical Trial Registration:**

ClinicalTrials.gov, identifiers NCT02794675 and NCT02467738.

## Introduction

The pattern of failure for head and neck squamous cell carcinoma (HNSCC) is classified as local, regional, or metastatic. Local/regional failure after treatment of primary HNSCC commonly arises, with rates ranging from 30 to 50% (1, 2). If the pattern of failure is local or regional, surgery with or without re-irradiation is the standard of care for resectable tumors (3). Conversely, for patients with unresectable disease, re-irradiation and/or systemic therapies are more frequently used treatment options. Systemic therapies, including chemotherapy and/or immunotherapy, remain the mainstay of treatment for metastatic disease. Based on this current standard, it is difficult to reconcile the high failure rate after salvage surgery alone with the additional morbidity and uncertain oncologic benefit of re-irradiation using external beam radiation therapy (EBRT). Therefore, surgery alone and surgery plus re-irradiation with intensity modulated radiation therapy (surgery + ReIMRT) pose major challenges.

In recurrent HNSCC, surgery alone typically provides poor and insufficient oncologic benefit, as demonstrated by an observed 2-year weighted average DFS of 36% and 5-year weighted average OS of 36.4% (range, 23-55%) in a meta-analysis of 343 patients treated with only salvage surgery (3). To address this poor prognosis, re-irradiation using EBRT techniques, specifically IMRT, is often added. In properly selected patients, there is a clear oncologic benefit to EBRT (4). However, if poorly selected patients are treated with EBRT, they are more likely to experience significant side effects and variable oncologic benefit, which has led to clinician hesitation when recommending re-irradiation with EBRT. It is important that clinicians understand the limitations of EBRT and select the correct patients for this treatment (1, 4–6).

In a study to compare re-irradiation using IMRT *versus* stereotactic body radiation therapy (SBRT), the unadjusted 2-year OS rate for IMRT was 35.4% and 16.3% for SBRT (P<0.01) (7). Lee et al. also showed the improved local/regional recurrence-free survival at 2 years was higher in IMRT (52%) compared to conventional radiation therapies (20%) (8). In a review of re-irradiation with EBRT in the post-salvage surgery setting, Strojan et al. observed a local/regional control rate of 13-74% (average: 38%) and OS rate of 21-67% (average: 43%) at two years (6). Despite this improvement in survival benefit, re-irradiation complications are significant and range from low grade, including infection, hematoma, hoarseness, and dermatitis to high grade, including fistula formation, osteoradionecrosis (ORN), tracheostomy dependence, wound dehiscence, esophageal stricture, and dysphagia requiring PEG tube placement (1, 9). Among many studies, Takiar et al. demonstrated re-irradiation with IMRT led to significant complications, reporting a 5-year grade ≥3 toxicity rate of 48% and three patient deaths due to radiation toxicity (1). Consequently, clinicians and researchers need to develop new ways of delivering therapeutic radiation doses to regions of head and neck disease, while minimizing treatment-related morbidity.

These issues have led to the use of brachytherapy, with and without salvage surgery, for local or regional recurrence of HNSCC. A recent review described 1,003 patients treated for recurrent HNSCC with Iridium-192 (Ir-192), Cesium-131 (Cs-131), and Iodine-125 (I-125) and found that brachytherapy, used in conjunction with surgical resection, enhanced regional control (reported 31% local/regional recurrence) compared to patients treated with brachytherapy alone (reported 49% local/regional recurrence) (9–12). Additionally, Henderson et al. demonstrated that low- and high-dose brachytherapy did not increase tissue flap morbidity after salvage treatment in patients with recurrent HNSCC (13).

The significant morbidity of re-irradiation in recurrent HNSCC drives the research behind alternative therapeutic options to enhance survival in patients with resectable, local/regional disease, while minimizing treatment-related morbidity. Theoretically, with the appropriate radioactive properties, brachytherapy could have numerous advantages over intensity-modulated radiation therapy (IMRT) (14). The novel use of Cs-131 in the treatment of HNSCC addresses some of the obstacles that have historically prevented the more widespread use of brachytherapy (15). Here, we present a multi-institutional clinical trial designed to prospectively enroll patients with local/regional HNSCC recurrence for Cs-131 placement at the time of surgical resection. Two retrospective cohorts from the four institutions were also reported, which included surgery plus re-irradiation with intensity modulated radiation therapy (surgery + ReIMRT) and surgery alone.

## Methods and Materials

This study was approved through respective institutional review boards from each participating institution. Subjects were enrolled in a prospective fashion under the approval of institutional IRBs (NCT02467738, NCT02794675).

### Patient and Tumor Characteristics

A multidisciplinary team approach was used to determine eligibility for Cs-131 brachytherapy. To qualify for enrollment in the prospective component of this trial, patients were required to have recurrent HNSCC with a previous history of radiation therapy and resectable disease. Patients were excluded if recurrence was not within the field of previous treatment. Subjects with evidence of metastatic disease were not eligible for enrollment. **Tables 1**, **2** provide demographic information.

**Table 1:** Demographic and tumor characteristics of the prospective cohort.

	*Prospective*
	Surgery + Cs-131
	(n=49)
**Age: Mean/Range**	65 (20-88)
**Gender: (Male : Female)**	32:17
**Primary Site**	
Cutaneous	5 (10%)
Larynx/Hypopharynx	8 (16%)
Oral Cavity	16 (33%)
Oropharynx	13 (27%)
Salivary Parotid	3 (6%)
Nasopharynx	2 (4%)
Unknown Primary	2 (4%)
**Recurrence Location**	
Local only	19 (39%)
Local and Regional	8 (16%)
Regional only	22 (45%)
**Treatment Prior to Recurrence**	
Surgery + adjuvant XRT +/- CRT	32 (65%)
Definitive XRT +/- CRT	17 (35%)
**Stage at Time of Recurrence, AJCC 7^th^ Edition**	n=31
I	2 (7%)
II	1 (3%)
III	6 (19%)
IV	22 (71%)
**HPV Positive (%)**	6 (12%)
**Perineural Invasion (%)**	29 (59%)
**Extracapsular Extension** (among pt w/ regional failure)	12 (55%)
	n=22
**Resection Margin (% positive)**	4 (21%)
(among pt w/ local failure)	n=19
**Smoking Status**	
Never	16 (34%)
Former	25 (53%)
Current	6 (13%)
**Alcohol Use**	
Never	24 (51%)
Former	3 (6%)
Current	20 (43%)

**Table 2:** Demographic and tumor characteristics of the institutional historic cohorts.

	*Institutional Historic Cohorts*	
	Surgery + ReIMRT	Surgery Alone
	(n=30)	(n=29)
**Age: Mean/Range**	59 (43-80)	62 (46-83)
**Gender: (Male : Female)**	20:10	23:6
**Primary Site**		
Cutaneous	2 (7%)	1 (3%)
Larynx/Hypopharynx	6 (20%)	12 (41%)
Oral Cavity	7 (23%)	3 (10%)
Oropharynx	13 (43%)	9 (31%)
Salivary Parotid	0 (0%)	1 (3%)
Nasopharynx	1 (3%)	1 (3%)
Unknown Primary	1 (3%)	2 (7%)
**Recurrence Location**		
Local only	10 (33%)	19 (66%)
Local and Regional	11 (37%)	5 (17%)
Regional only	9 (30%)	5 (17%)
**Treatment Prior to Recurrence**		
Surgery + adjuvant XRT +/- CRT	9 (30%)	11 (38%)
Definitive XRT +/- CRT	21 (70%)	18 (62%)
**Stage at Time of Recurrence, AJCC 7^th^ Edition**	n=29	n=28
I	1 (3%)	2 (7%)
II	1 (3%)	1 (4%)
III	2 (7%)	3 (11%)
IV	25 (86%)	22 (79%)
**HPV Positive (%)**	7 (23%)	6 (21%)
**Perineural Invasion (%)**	15 (50%)	17 (59%)
**Extracapsular Extension **(among pt w/ regional failure)	6 (67%)	2 (40%)
	n=9	n=5
**Resection Margin (% positive) **(among pt w/ local failure)	2 (20%)	4 (21%)
	n=10	n=19
**Smoking Status**		
Never	4 (13%)	7 (24%)
Former	19 (63%)	15 (52%)
Current	7 (23%)	7 (24%)
**Alcohol Use**		
Never	14 (47%)	9 (32%)
Former	0 (0%)	6 (21%)
Current	16 (53%)	13 (46%)

**Figure 1:**
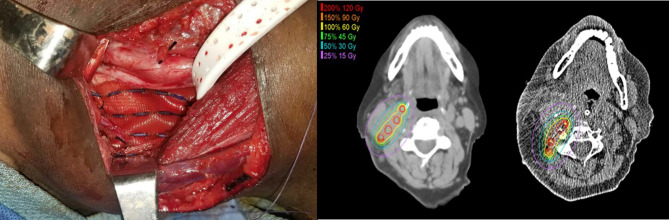
Intraoperative adjustments were made for placement around major vessels, nerves, and bone. Components of the harvested flap were utilized to avoid direct contact of the Cs-131 seeds with critical structures, including the carotid artery.

Preplanning was completed by a head and neck cancer surgeon and radiation oncologist to determine the total area of risk to be covered, the prescribed radiation dose, the strength and number of the Cs-131 seeds, and the surrounding critical structures of concern. Cs-131 seeds were ordered prior to surgical resection (16). Source placement followed conventional planar implant techniques. Uniform-activity seeds were ordered in individual strands or a pre-loaded mesh. Seeds were stranded 10 mm apart, with the intent to implant individual strands 10 mm apart. If a mesh was ordered, seeds were sewn into the mesh in a 10 mm by 10 mm grid pattern. All seeds were implanted in a single plane, and dose was prescribed to a point 5 mm from the implant plane. Seed activity was selected such that 60 Gy was delivered to a depth of 5 mm from the center of the source plane. The number of seeds required for an implant was determined by the estimated size of the future post-resection cavity; sufficient seeds were implanted in the entire resection cavity to deliver the prescribed dose (16). After gross total resection of the tumor and harvesting of any flap required for reconstruction, the Cs-131 seeds were implanted into the surgical bed, either as free strands or as a mesh. Intraoperative adjustments were made for placement around major vessels, nerves, and bone, and components of the harvested flap were utilized to avoid direct contact of the Cs-131 seeds with critical structures (**Figure 1**). Postoperatively, patients underwent non-contrasted CT scan, and actual post-implant dosimetry was calculated after determining the final seed position.

The prospective trial was designed as a phase 1/2 pilot trial with safety as the primary endpoint. Adverse events (AEs) were collected utilizing the Common Terminology Criteria for Adverse Events (CTCAE) version 5.01; both acute (within 30 days) and long-term complications (reported through most recent follow-up) were recorded. In the prospective cohorts, AEs were documented at three-month intervals for two years. In the retrospective cohorts, AEs were recorded from the patients’ electronic health records. Percutaneous endoscopic gastrostomy (PEG) tube and tracheotomy dependence were recorded for each cohort. Re-occurrence, death, and cause of death after completion of therapy were also collected. Secondary endpoints involving oncologic outcomes included three time-to-event endpoints: local/regional disease-free survival (DFS), overall DFS, and overall survival (OS). Surgery and/or Cs-131 placement occurred at the site of primary recurrence (local) or in the neck (regional). There were no local or regional failures outside the area of treatment. Metastatic disease was not considered recurrence-related to the treatment cohorts when calculating DFS.

Two additional cohorts—patients who underwent surgery alone or surgery + ReIMRT—were collected retrospectively from the institutions during the same time period as the prospective trial enrollment, as a matter of reference. Patient demographics for these cohorts are listed in **Tables 1**, **2**. Subjects in these institutional historic cohorts were required to have prior EBRT and recurrence of previously treated HNSCC. The inclusion/exclusion criteria were the same as those used for enrollment into the prospective surgery + Cs-131 cohort.

### Statistical Analysis

The demographics and AEs are summarized using means, standard deviations, and percentages. The three time-to-event endpoints (local/regional DFS, overall DFS, and OS) are summarized with the 95% confidence bands (17). All analyses were performed using R 4.0.0 (R Core Team, 2020) software, including the following packages: *survival* and *coxphw* (18, 19).

## Results

### Prospective Cohort (Surgery + Cs-131)

Forty-nine patients were enrolled in the surgery + Cs-131, in which just over half (53%) had a former smoking history and 12% were oropharyngeal HPV positive (**Table 1**). At the time of recurrence, almost all patients were diagnosed with stage III (19%) or stage IV (71%) disease (AJCC 7^th^ edition).

At base line, 31% of patients underwent tracheostomy, and 20% had a PEG tube placed at the time of surgery or up to 2 years after surgery + Cs-131. Treatment-related AEs are summarized in **Table 3**, which reports that 24% of surgery + Cs-131 patients experienced wound or vascular complications. One case of a carotid artery blow-out, unrelated to Cs-131, occurred secondary to a wound complication, and a subsequent decision was made to transition the patient to hospice care. The patient had a prescribed dose of 60 Gy of Cs-131 with seed activity of 1.8 U and 38 seeds implanted in the submental region. The minimal distance was 1.5 cm with a carotid D_max_ (1 mm^3^) of 12 Gy (11-12 Gy) and a carotid D_2cc_ of 4 Gy (2-6 Gy). There were no reports of ORN in this cohort, extending out 3 years. The majority (57%) of patients had grade 0 treatment-related AEs, whereas 37% experienced grade 1 to 3 AEs, and 2 patients suffered from grade 4 treatment-related AEs **(** **Table 3** **)**.

**Table 3:** Adverse events.

	*Prospective*	*Institutional Historic Cohorts*	
	Surgery + Cs-131	Surgery + ReIMRT	Surgery Alone
	(n=49)	(n=30)	(n=29)
**Tracheostomy**	15 (31%)	13 (43%)	8 (27%)
**PEG due to Treatment for Recurrence**	10 (20%)	18 (60%)	11 (38%)
**Wound Complications**	9 (18%)	10 (33%)	10 (35%)
**Vascular Complications**	3 (6%)	1 (3%)	4 (14%)
**Osteoradionecrosis**	0 (0%)	5 (17%)	0 (0%)
**Grade of Treatment-Related AE**			
**0**	28 (57%)	4 (13%)	9 (31%)
**1-3**	18 (37%)	25 (83%)	19 (66%)
**4-5**	3 (6%)	1 (3%)	1 (3%)

To determine the oncologic outcomes of surgery + Cs-131, DFS and OS were examined (**Table 4**). Recurrence after completion of treatment was designated as local, regional, local/regional, or metastatic. The KM estimated the five-year local/regional DFS rate was 49% (95% CI, 32-73%). Of the 19 patients with local disease at time of entry into the study, the 2-year local DFS rate was found to be 38% (95% CI, 20-71%). Of the 22 patients with only regional (neck) disease at the time of entry into the study, the 2-year regional DFS rate was observed to be 64% (95% CI, 39-100%) Thirty-one percent of patients experienced post-treatment metastasis, driving down the OS to 31% (95% CI, 15-63%) at 5 years while maintaining a local/regional DFS rate of 49% (95% CI, 32-73%).

**Table 4:** Oncologic outcomes.

		Local/Regional DFS (2-year)	Local DFS	Regional DFS	Post-Treatment Metastasis Rate	Overall DFS Rate (5-year)	Overall Survival Rate (5-year)
*Prospective*	**Surgery + Cs-131**	49% (95% CI, 32-73%)	38% (95% CI, 20-71%)	64% (95% CI, 39-100%)	31%	24% (95% CI, 12-47%)	31% (95% CI, 15-63%)
*Institutional Historic Cohorts*	**Surgery + ReIMRT**	61% (95% CI, 44-83%)	44% (95% CI, 21-92%)	65% (95% CI, 39-100%)	23%	29% (95% CI, 16-55%)	37% (95% CI, 22-62%)
	**Surgery Alone**	40% (95% CI, 24-66%)	39% (95% CI, 21-70%)	33% (95% CI, 7-100%)	31%	17% (95% CI, 5-51%)	17% (95% CI, 3-81%)
*Historical*	**Surgery Alone** (Goodwin WJ) (1)	–	–	–	–	36.3% (2-year DFS)	36.4% (range, 23-55%)
	**Surgery + ReIMRT** (Takiar et al.) (4)	–	–	–	–	–	57%

### Institutional Historic Cohorts (Surgery + ReIMRT and Surgery Alone)

Our institutional historic cohorts included 59 patients, of which, 30 were treated with surgery + ReIMRT, and 29 were treated with surgery alone (**Table 2**). Adverse events at the time of treatment were also reported for both cohorts (**Table 3**). Patients in the surgery alone cohort experienced fewer low-grade (grade 1-3) AEs (66%) than the surgery + ReIMRT cohort (83%). The rate of PEG tube placement due to salvage treatment (defined as a PEG tube placed any time during or after salvage surgery) was significantly lower in the surgery alone (38%) cohort compared to the surgery + ReIMRT (60%) group (p = 0.002). The rate of tracheostomy procedures was also lower in surgery alone (38%) compared to surgery + ReIMRT (43%). Wound complication rates were similar in surgery + ReIMRT (33%) and in surgery alone (35%), as re-irradiation was not a factor in postoperative complications due to planned delivery 4-6 weeks after surgery. Based on treatment records, ORN was observed only in the surgery + ReIMRT cohort (17%) and did not occur in patients who underwent surgery alone (p = 0.002).

Despite the higher complications rates seen in the surgery + ReIMRT cohort, the KM estimated two-year local/regional DFS rate for surgery + ReIMRT was 61% (95% CI, 44-83%), while the rate for surgery alone was 40% (95% CI, 24-66%) Comparatively, the KM estimated five-year local/regional DFS rate for surgery + ReIMRT was 52% (95% CI, 33-80%), and surgery alone was 27% (95% CI, 10-69%).

## Discussion

The varied oncologic benefits, inadequate delivery methods, and significant treatment-related adverse effects of therapies for local/regional HNSCC have led to the exploration of interstitial brachytherapy in recurrent HNSCC. Unfortunately, brachytherapy use still remains limited, which may be due to inexperience with brachytherapy implantation techniques and/or concerns of radiation exposure to hospital personnel (20). However, multiple publications have reported the success and benefits of brachytherapy (2, 21–24). Glatzel et al. showed an overall remission rate of 81% in recurrent. HNSCC using high-dose brachytherapy with only 6.7% of patients experiencing grade 3 and 4 toxicity (21).

Rodin et al. also performed preliminary analysis across multiple studies that showed the rate of local/regional recurrence was 38.8% in surgery + brachytherapy *versus* 49% in brachytherapy alone (odds ratio: 0.66; 95% CI: 0.38-1.12; p = 0.13) (9). Compared to EBRT, brachytherapy uses lower energy photons for localized dose delivery, decreasing the radiation distribution to healthy tissues and the potential for subsequent treatment-related complications to allow for better quality of life (9, 16, 25). The delivery of brachytherapy can be administered using a removable catheter or permanent insertion during surgery. The novel use of Cs-131 in the treatment of HNSCC addresses some of the obstacles that have historically prevented the more widespread use of brachytherapy, which include a half-life profile that allows for delivery of the therapeutic dose within approximately 40 days, minimal isolation precautions, and permanent implantation (9). As seen in this prospective study, Cs-131 brachytherapy was permanently placed to provide a suitable therapy option for recurrence with irregular surfaces and eliminate the need for removal (9, 25).

Kharouta et al. published their prospective study of 12 patients with 2-year follow-up to investigate the safety of surgery and permanent Cs-131 brachytherapy placement in high-risk recurrent head and neck cancer (26). Based on the small cohort of patients who received surgery + Cs-131, no serious adverse events were reported, and only 2 patients experienced wound breakdown with subsequent need for local wound care (26). The overall survival at 1- and 2-years was 75% and 58%, respectively, with 3 patients having local/regional recurrence in the site of Cs-131 placement (26). These results point to encouraging safety and efficiency outcomes of surgery + Cs-131 treatment for recurrent HNSCC. Our study results, which includes 49 patients from multiple institutions followed over 5 years, strengthens and supports the findings of Kharouta.

Our study reports the results of a multi-institutional prospective phase 1/2 trial for local and regional recurrence of HNSCC treated using surgery with adjuvant Cs-131 brachytherapy to understand its safety and oncologic therapy profile. Although statistical comparisons and conclusions cannot be drawn from historic controls, we present them together as a comparative baseline. In the surgery + Cs-131 cohort, the rates of tracheostomy (31%), PEG tube placement (20%), and wound complications (18%) were significantly lower than the reported institutional historic cohort findings of surgery + ReIMRT complications (43%, 60%, and 33%, respectively). No cases of ORN occurred in the surgery + Cs-131 cohort, and the majority of treatment-related AEs were grade 0. The one patient who suffered from a carotid rupture, secondary to wound complications, only had 12 Gy of radiation delivered to the carotid, which was calculated based on post-implant dosimetry. The two-year local/regional DFS was 49% for the patients receiving surgery + Cs-131 *versus* a regional DFS of 64%. These numbers are comparable to surgery + ReIMRT, with a 61% local/regional DFS (**Table 3**). Surgery alone demonstrated regional DFS in only 33% of subjects at two years. The OS at 5 years was poor across all cohorts, driven largely by post-treatment metastatic disease, which resulted in 31% in surgery + Cs-131, 37% in surgery + ReIMRT, and 17% in surgery alone.

Although surgery + Cs-131 treatment demonstrates promising results, it is important to recognize that our prospective study and the Kharouta et al. study do not address the metastatic pattern of failure in HNSCC and prompts the need for more holistic approaches (26). Systemic treatment, such as the immunotherapy PD-1 or PD-L1 checkpoint inhibitors, has the potential to reduce the risk of relapsing and metastatic disease. A recent phase II trial utilizing pembrolizumab in the neoadjuvant and adjuvant setting with surgery was safe and effective in recurrent and metastatic HNSCC (27). A pathologic response was observed in 44% of trial patients, and the 1-year relapse rate was lowered compared to historical data (27). Clinicaltrial.gov reports one ongoing single-arm, multi-institutional phase 1b/2 study that combines the use of perioperative pembrolizumab, salvage surgery, and Cs-131 brachytherapy to improve metastatic HNSCC disease control (28). This combination therapy trial is a suitable strategy to address some of the challenges that metastatic patterns of failure in HNSCC pose.

Our study design intentionally had a broad inclusion criterion for the prospective cohort, which encompassed a range of head and neck pathologies, including cutaneous and parotid gland lesions and mucosal squamous cell carcinomas, with the intent to establish a safety endpoint. Future trials, including the combination of Cs-131 and immunotherapy (NCT04340258), will narrow the inclusion criteria. Limitations of this trial are highlighted by the lack of prospective control groups, preventing statistical comparison of all three cohorts. Lastly, bias may have been introduced due to the multiple participating institutions. In an effort to control for individual surgeon bias and institutional bias, surgical protocols were outlined for Cs-131 placement, and an interdisciplinary team was consistently involved across all participating institutions. Institutional treatment patterns for recurrent HNSCC were also used to the study’s advantage.

## Conclusions

This prospective trial demonstrates that the use of intraoperative Cs-131 brachytherapy during salvage surgery for recurrent HNSCC has an acceptable safety profile, including lower rates of ORN and PEG tube placement, compared to historic cohorts of re-irradiation after surgery. Importantly, local/regional 2-year DFS in the surgery + Cs-131 was acceptable and similar to both retrospective and historical groups. Additional randomized studies are needed to determine non-inferiority patterns of practice in patients with recurrent HNSCC.

## Data Availability Statement

The raw data supporting the conclusions of this article will be made available by the authors, without undue reservation.

## Ethics Statement

The studies involving human participants were reviewed and approved by Thomas Jefferson University IRB. The patients/participants provided their written informed consent to participate in this study.

## Author Contributions

Conceptualization: AL, DK, CZ, TW-D, VK, JJ, WK, JC, DC, VB-A. Data Curation: AL, DK, CZ, TW-D, VK, JJ, WK, JC, DC, VB-A, AC, JP, MC, BP, SG, MY, PL, VT, AT. Formal Analysis: TZ, JP, MC, AC. Investigation: BP, SG, MY, PL, VT, AT. Methodology: AL, DK, CZ, TW-D, VK, JJ, WK, JC, DC, VB-A. Resources: AL, DK, CZ, TW-D, VK, JJ, WK, JC, DC, VB-A, BP, SG, MY, PL, VT, AT. Supervision: AL, DK, CZ, TW-D, VK, JJ, WK, JC, DC, VB-A. Validation: AL, DK, CZ, TW-D, VK, JJ, WK, JC, DC, VB-A, AC, JP, MC. Writing-original draft preparation: AL, DK, CZ, TW-D, VK, JJ, WK, JC, DC, VB-A, AC, JJ, BP, SG, MY, PL, VT, AT. Writing – review and editing: BP, SG, MY, Pl, VT, AT. All authors contributed to the article and approved the submitted version.

## Conflict of Interest

The authors declare that the research was conducted in the absence of any commercial or financial relationships that could be construed as a potential conflict of interest.

## Publisher’s Note

All claims expressed in this article are solely those of the authors and do not necessarily represent those of their affiliated organizations, or those of the publisher, the editors and the reviewers. Any product that may be evaluated in this article, or claim that may be made by its manufacturer, is not guaranteed or endorsed by the publisher.
